# Barriers and motivators to undertaking physical activity in adults over 70—a systematic review of the quantitative literature

**DOI:** 10.1093/ageing/afae080

**Published:** 2024-04-22

**Authors:** Alixe H M Kilgour, Matthew Rutherford, Joanna Higson, Samantha J Meredith, Jessica McNiff, Stephanie Mitchell, Anusan Wijayendran, Stephen E R Lim, Susan D Shenkin

**Affiliations:** Ageing and Health Research Group, Usher Institute, University of Edinburgh, UK; University of Edinburgh Medical School, UK; Department of Medicine of the Elderly, NHS Lothian, UK; Academic Geriatric Medicine, NIHR Applied Research Collaboration Wessex, University of Southampton, UK; Department of Medicine of the Elderly, NHS Lothian, UK; Department of Medicine of the Elderly, NHS Lothian, UK; University of Edinburgh Medical School, UK; Academic Geriatric Medicine, NIHR Applied Research Collaboration Wessex, University of Southampton, UK; Ageing and Health Research Group, Usher Institute, University of Edinburgh, UK; Advanced Care Research Centre, Usher Institute, University of Edinburgh, UK

**Keywords:** barriers, facilitators, physical activity, older adults, systematic review, older people

## Abstract

**Background:**

Physical activity (PA) has multiple benefits for older adults (≥70 years old). Despite this many older adults do not undertake the World Health Organisation guideline recommended amount of PA. This systematic review examines barriers and motivators to PA in adults aged ≥70 years.

**Methods:**

We analysed the quantitative literature, including observational studies and baseline data from randomised controlled trials. Studies examining specific diseases (e.g. cognitive impairment), or care home residents were excluded. Database searches of ASSIA, CINAHL, Embase, Medline, PsycINFO and Web of Science were undertaken on 7 March 2023. Quality assessment was performed using the ROBANS tool. We synthesised the results using the socioecological model. The protocol was registered on PROSPERO (CRD42021160503).

**Results:**

We identified 37 papers, *n* = 26,961, age 70–101 years (median 78), 62% female. We undertook a narrative review; meta-analysis was not possible. Overall risk of bias was low. A total of 23 studies addressed barriers, seven motivators, seven both. The most cited barriers were: concern about physical health/fitness (14 studies), lack of motivation/interest (13 studies), fear of falls/history of falling (11 studies) and environmental barriers (10 studies). Key motivators were: support from family/friends (five studies), social interaction (five studies), personal benefits (five studies) and outside facilities (five studies). Results varied across gender, age, functional ability and geographical location.

**Discussion:**

To maximise PA in older adults, important modifiable factors identified in this review should be targeted: support from healthcare professionals; reducing fear of falls; and prioritising ease of access and safety of outdoor facilities. When considering future policy, a person-centred, age group appropriate approach will have the most impact.

## Key Points

- Barriers and motivators to physical activity vary according to gender, age, functional ability and geographical location.
- A person-centred approach is key when formulating individualised advice to increase physical activity.
- Key barriers are fitness and health; motivation/interest; fear of falling/history of falling; and environmental barriers.
- Key motivators are support from family and friends; social interaction; personal benefits; and outside facilities.
- Three modifiable factors to promote physical activity were identified which can inform future policy and practice.

## Background

Physical activity (PA) reduces the incidence of sarcopenia [1], frequency of falls [2], cardiovascular disease [3], days spent in hospital [4], and all-cause mortality [5], even in frail older adults. The World Health Organisation (WHO) recommends that older adults undertake a minimum of 150 min of moderate aerobic PA per week, plus activity to build strength and balance 3 days a week [6]. Despite the well-documented benefits of PA, the number of older adults achieving the recommended weekly target is lower than younger age groups [7]. In the 2016 Health Survey for England only 36% of men and 26% of women over the age of 75 met the guidelines for aerobic PA [8], and similarly in the 2019 Scottish Health Survey only 41% of men and 31% of women [9]. If we include muscle strengthening and balance exercises, only 10% of men and 6% of women in Scotland over the age of 75 meet the current recommendations [9].

The proportion of people over the age of 70 in the world is estimated to more than treble from 6.4% to 20.8% by 2100, therefore the potential public health benefit from increasing PA in this age group is clear [10]. Identifying common barriers and motivators for older adults is a key step in the development of strategies to promote PA. Previous systematic reviews have focussed on younger age groups [11, 12], included both younger and older adults [13], or included older age groups in the context of a specific morbidity or demographic [14, 15]. This systematic review aims to examine the quantitative literature detailing barriers and motivators to PA in adults over the age of 70.

**Figure 1:**
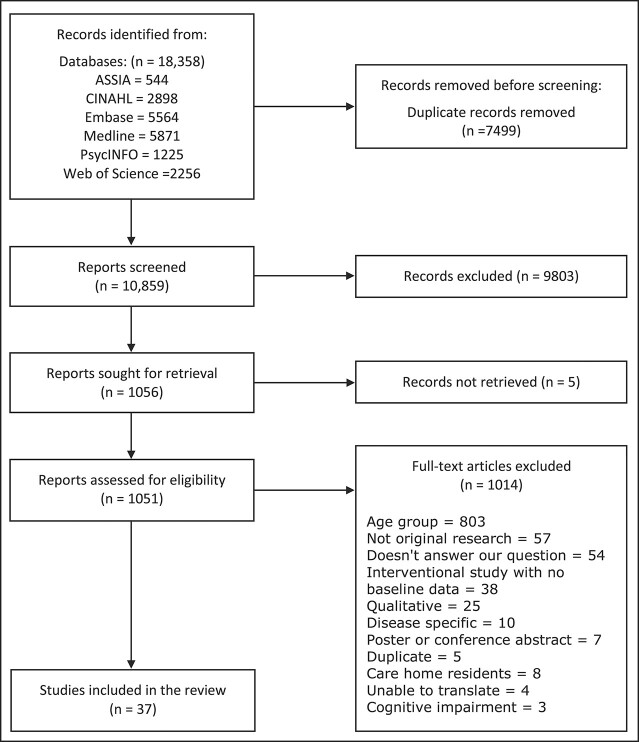
PRISMA 2020 flow diagram summarising study inclusion.

## Methods

The systematic review protocol was registered on PROSPERO (CRD42021160503) and is reported using PRISMA guidelines.

### Inclusion and exclusion criteria

Inclusion criteria were (i) participants aged ≥70 years (this age cut-off was chosen due to the increased prevalence of frailty, sarcopenia, and assistance required with activities of daily living (ADLs) over 70 [16–18]), (ii) observational studies of barriers and motivators to PA (comprising cohort and cross-sectional studies, randomised controlled trials where baseline data were available and case control studies where control group data were available) and (iii) any amount or intensity of PA, with PA defined by WHO as ‘any bodily movement produced by skeletal muscles that requires energy expenditure’ [6]. This report includes the quantitative studies (i.e. methodology generating numerical results), and those with mixed methods that report quantitative results.

Exclusion criteria were (i) studies examining a specific disease group (e.g. post-stroke), (ii) care home residents, defined as ‘a facility with a domestic-styled environment that provides 24-h functional support and care for persons who require assistance with ADLs and who often have complex health needs and increased vulnerability’ [19], (iii) studies with a focus on participants with cognitive impairment, (iv) grey literature (e.g. conference abstracts) and (v) non-English studies if there was no available translator in our team (studies written in French, German and Spanish were included).

### Search strategy

Database searches of ASSIA, CINAHL, Embase, Medline, PsycINFO and Web of Science were devised by two researchers (AW, JH) and a university librarian, and were undertaken from inception until 7 March 2023. The search strategies are shown in appendix S1. All languages were included in the search.

### Study selection

Each paper was screened independently by two of four authors (JH, JM, SM, AW). In a separate process, the full text of the short-listed studies was then reviewed independently by any combination of two of four authors (JH, JM, SM, MR), and reasons for exclusion were recorded. Disagreements were resolved by discussion with a senior author (AK).

### Data extraction

Participants’ demographics, aim of the study, study type, method of data analysis and key results related to barriers and motivators to PA were recorded by three researchers (JH, AK, MR) using a data extraction form (appendix S2).

### Data synthesis

We hoped to undertake a meta-analysis, but due to the heterogeneity of the predictor and outcome variables between the studies the results were not comparable enough (see Table 1). Furthermore, most studies did not report an effect size, solely providing the percentage of participants reporting a factor, or the rank order of a list of barriers and/or motivators.

**Table 1:** **Overview of included studies** (ordered alphabetically within country of origin)

**Reference**	***n***	**Country of origin and study**	**Age**	**% Female**	**Study type**	**Primary outcome**	**Main results**
Booth, Bauman & Owen, 2002 [48]	153	**Australia**, 1995 Population Survey Monitor	70+	57.9	Cross-sectional study	Barriers to PA	• Most commonly reported barriers: injury/disability (39.6%), already fit enough (37.9%) and ‘too old’ (36.2%) • No respondents found the following items a barrier: ‘I have not got the right clothes or equipment’ and ‘I’d never keep up’
Brown & Miller, 2001 [49]	395	**Australia**, Australian Longitudinal Study on Women’s Health	72–79	100	Longitudinal cohort study	Barriers to PA in women- specifically urine leakage	• The only study to enquire about urine leakage as a barrier • 15.9% of older women reported leaking urine in the last month whilst playing sport or exercising • 27.5% avoided sporting activities in the past month because of urine leakage
Bruce, Devine & Prince, 2002 [50]	1,500	**Australia**	70–85 Mean 75.2 (SD 2.7)	100	Baseline data for RCT	Barriers to PA- specifically fear of falling	• Fear of falls (OR 0.70 (0.53–0.90)) was predictive of being sedentary but history of falls was not • Mean energy expenditure was 183 kcal/day in women reporting fear of falls, and 216 kcal/day in those who did not report a fear of falls
Kirkby et al., 1999 [39]	45	**Australia**	75+	100	Cross-sectional study	Motivators for exercise	• Top three ranked reasons for participating in exercise were enjoyment of exercise, wanting to improve physical fitness and social aspects • The lowest ranked factors were related to popularity (e.g. I want to be popular ranked last)
Newson & Kemps, 2007 [51]	94	**Australia**	75+	Not given	Cross-sectional study	Motivators and barriers to exercise	• Barriers in order of importance (factor loadings): medical (21.6%), concern (16.8%), situational (14.3%), facilities/knowledge (13.3%)
							• Motivators in order of importance (factor loadings): fitness (28.2%), social engagement (14.3%), challenge (10.7%), health (9.9%)
Hamm et al., 2014 [33]	107	**Canada**, Ageing in Mantoba (AIM) study	80–97 Mean 83.86 (SD 3.95)	68	Prospective cohort study	How primary and secondary control relate to PA	• Having both high primary and secondary (i.e. behaviours targeted at internal processes to aid levels of primary control) control was significantly linked to increased everyday PA compared with conflicted engagement (i.e. low primary control and high secondary control) (770 vs 573 counts on actigraph, *P* < 0.05)
Cousins, 1995 [35]	327	**Canada**	70–98 Mean 76.7 (SD 5.5)	100	Cross-sectional study	Sources of encouragement and personal attributes, and exercise	• Better social support was predictive of higher exercise level • Having friends interested in physical fitness activities had the strongest relationship with PA level (β 0.32, *P* < 0.001)
Cousins, 1996 [34]	327	**Canada**	70–98 Mean age 76.7 (SD 5.5)	100	Cross-sectional study	Cognitive variables as determinants of late life exercise	• Social support (β 0.35, *P* < 0.01) and self-efficacy (movement confidence) (β 0.28, *P* < 0.01) were significantly associated with exercise level • Health incentive, perceived risks and benefits of exercise, and health locus of control score were not (*P* > 0.01)
Eronen et al., 2014a [45]	261	**Finland**, Screening and Counseling for Physical Activity and Mobility in Older People (SCAMOB)	75–81 Mean age 77.7	73.4	Control group of RCT	Effect of environmental facilitators on the development of walking difficulty	• The commonest environmental facilitators for outdoor walking were park or other outdoor area within walking distance (93.5%); outdoor recreational facilities within walking distance (92%) and attractive features in the nearby environment (64.8%) • Having all five measured environmental facilitators was associated with a decreased risk of developing walking difficulty at 3.5 year follow-up (HR 0.41, CI 0.21–0.84)
Eronen et al., 2014b [42]	848	**Finland**, Life-Space Mobility in Older People (LISPE)	75–91 Mean 80.1 (SD 4.3)	62	Baseline data from cohort study	Barriers to PA and evaluating unmet PA need	• Latent class modelling identified five different profiles for outdoor activity: 46.5% Minor barriers; 26.5% Ambient conditions; 15.9% Poor health; 6.5% Mobility limitations; and 4.6% Insecurity
							• There was wide variation in reporting of barriers between groups: slippery roads was reported as a barrier by 0% in the Minor barriers profile and 97% in the Mobility limitations profile • 1.4% of those in the Ambient conditions profile found lack of motivation a barrier, rising to 15.4% of those in the poor health profile
Keskinen et al., 2020a [43]	167	**Finland**, LISPE	74.3–89.3 Mean 80.3	65	Cohort study	Associations of environmental features with outdoor PA on weekdays and weekends	• Number of bouts of PA and time spent in moderate to vigorous PA did not vary between weekday versus weekend
							• Environmental features (e.g. habitat diversity) were associated with more PA on weekdays but not at weekends • High population density was associated with 0.21 more PA bouts and 23 more minute of PA per day than the low population density
Keskinen et al., 2020b [44]	848	**Finland**, LISPE	75–90 Mean 80.6	62	Cohort study	Impact of neighbourhood type on PA level and perceived facilitators to PA	• Residents of high population density areas were 2–4x more likely to undertake at least moderate PA if they perceived nature-based facilitators, whereas residents of low-density areas were 2x more likely to undertake at least moderate PA if they perceived certain infrastructure-based facilitators
Portegijs et al., 2017 [22]	848	**Finland**, LISPE	75–90 Mean 80.6 (SD 4.3)	64	Baseline data from cohort study	Entrance-related environmental barriers and daily out of home mobility	• Multiple objectively measured barriers at the entrance of a dwelling (e.g. stairs) was associated with an increased chance of leaving the house less than daily (OR 1.8, 95% CI 1.04–3.1), but having barriers in the exterior surroundings (e.g. narrow path) (*P* > 0.05) was not • Perceived barriers at the entrance of a dwelling was also associated with an increased chance of leaving the house less than daily (OR 1.6, 95% CI 1.01–2.5)
Rantakokko et al., 2010 [46]	643	**Finland**, SCAMOB	75–81 Mean 77.6 (SD 1.9)	75	Baseline data from RCT, and control group for follow-up data at 2 years	Unmet PA need	• All the environmental barriers included in the analyses were associated with unmet PA need at baseline • Lack of resting places was more commonly reported by those with unmet PA need (OR 2.9, 95% CI 1.65–5.25) • Noisy traffic (OR 4.5, CI 1.65–12.16) and fear of moving outdoors (OR 3.1, CI 1.21–7.81) at baseline conferred significant risk of developing unmet PA need over 2 year follow-up, whereas poor street conditions, long distances, lack of resting places and dangerous crossroads were not
							• Living alone decreased the probability of having unmet physical need (OR 0.32, 95% CI 0.18–0.57)
Rantakokko et al., 2012 [32]	214	**Finland**, SCAMOB	75–81 Mean 77	78.5	Control group of RCT to 3.5 years follow-up	Perceived barriers in the outdoor environment	• Reporting lack of resting places and long distances at baseline doubled the risk of difficulty in walking 2 km (HR 2.19, 95% CI 1.31–3.64) and 0.5 km (HR 1.90, 95% CI 1.31–3.64) at 3.5 year follow-up • Reporting terrain and traffic as barriers at baseline did not predict incident difficulty in walking at 3.5 year follow-up
Rasinaho et al., 2007 [52]	645	**Finland**, SCAMOB	Mobility limitationmean (SD) Severe 77.9 (2.0) Moderate 77.9 (2.1) None: 77.4 (1.9)	35.7	Baseline data from RCT	Motives for and barriers to PA	• Severe mobility limitation vs no mobility limitation reported barriers: poor health (84% vs 24%, *P* < 0.001), fear and negative experiences (49% vs 13%, *P* < 0.001) and unsuitable environment (41% vs 25%, *P* < 0.001) • Lack of knowledge about exercise was rated the least important factor across all mobility levels
							• The most reported motivators in the severe limitation group were positive experience (e.g. exercise is uplifting) and knowledge about benefits (both 70%), whereas it was nearby facilities in the no limitation group (85%)
Sakari et al., 2017 [40]	848	**Finland**, LISPE	75–90	62.1	Baseline data from cohort study	Associations between perceived environmental and individual characteristics and walking limitations	• Most reported barrier was snow/ice in the winter, reported by 43% with no mobility limitations and 68% of those with advanced limitations • Seeing other walkers’ example inspired older adults to start recreational walking themselves, across all three mobility levels
							• Most reported environmental facilitator was being in nature (e.g. lakesides)
							• Importance of individual barriers and facilitators varied according to physical abilities
Tsai et al., 2013 [23]	657	**Finland**, SCAMOB	75–81 Mean 77.6 (SD 1.9)	75	Baseline data from RCT	Environmental mobility barriers and walking for errands	• Living with others was associated with being in the lowest distance walked per week for errands group, compared with living alone (24% v 8%) • For individuals living alone, barriers at dwelling entrance was associated with an 8x increased likelihood of walking <1.5 km per week for errands • For individuals living with others, distance-related barriers correlated to a 30 times increased incidence of walking <1.5 km per week for errands
Ferrand, Martinent & Bonnefoy, 2014 [36]	100	**France**	70+ Mean 75.34 (SD 4.89)	57	Cross-sectional study	Motivational profiles for exercise related to health-related quality of life (HRQoL)	• Participants in the highly self-determined profile exercised for more minutes per week than the moderately introjected group (e.g. guilt at not exercising) (464 vs 387 min per week, *P* = 0.02) • Intrinsic motivation (e.g. self-determined motivation) is a greater determinant of higher PA levels in older adults who exercise than extrinsic regulation (e.g. undertake exercise to receive obtain some sort of reward)
Benzinger et al., 2014 [24]	36	**Germany**	80+ Median 83.5	48.1	Cross-sectional study	Barriers to PA- using Person-Environment fit (PE fit)	• The magnitude of Person-Environment fit (P-E fit)* problems was significantly correlated with PA in older individuals (r_s_ = −0.388, *P* = 0.04) • Functional limitations explained 19.6% of the variance in PA, PE-fit score 11.1% and environmental barriers only 2.5%
Moschny et al., 2011 [53]	286	**Germany**, getABI study	72–93 Median 77	50.7	Cohort study	Barriers to PA in those who self-reported as not sufficiently active	• Poor health was the most reported barrier (57.7%), with the over 80s more likely to report it than 72–79 year age group (71.1% vs. 51.5%, *P* = 0.002), and lack of company was the second most reported barrier (43%) • Lack of time was the least important barrier (16.4%) • Women rated lack of transport (OR 5.3, 99.7% CI 1.8–15.7) and lack of opportunity (OR 2.4, 1.0–5.7) higher than men
Harada et al., 2018 [25]	2,824	**Japan**, National Center for Geriatrics and Gerontology	70+ Mean 75.6 (SD 4)	51.9	Cohort study	Expectation for PA to minimise dementia risk and measured PA level	• 85.8% of participants had at least some expectation for PA to decrease dementia risk • Neither expectation for decreased dementia risk nor perceived value of dementia prevention was correlated with increased PA
Aspvik et al., 2018 [26]	1,219	**Norway**, Generation 100 study	70–77	51	Baseline data from RCT	Weather changes and PA	• Weather explained 1.2% of the variance within older adults’ PA • Older adults had a higher PA level in warmer (597CPM) than colder months (556CPM) (*P* < 0.01). • As precipitation increased, PA of females decreased (-30CPM, *P* < 0.01) but there was no significant effect in males, and PA decreased in unfit individuals (-47CPM, *P* < 0.01), but there was no effect on moderately/highly fit individuals.
Sorensen & Gill, 2007 [27]	690	**Norway**, National Institute of Public Health 2000–2001	All age 75 in this subgroup	49.1	Cohort study	Perceived barriers to PA	• Women rated the health factor highest (mean score 1.45, barriers scored 0–3), then affective/cognitive (1.24), then priority (1.03), and least important were practical barriers (e.g. transport, cost) (0.87) • Men rated the factors in the same order of importance as women: health (1.30), affective/cognitive (1.21), priority (0.88) and lastly practical (0.59)
De Roza et al., 2023 [58]	163	**Singapore**	75+	66.6	Cross-sectional mixed methods study	Barriers to PA during COVID-19	• 30.1% of those 75+ felt unsafe to exercise during the pandemic, compared with 18.1% of those 65–74 years old (*P* = 0.30) • Concerns included wearing a mask during exercise; centres closing and formal sessions being stopped; family concerns and dislike of home-based exercise
Izquierdo Campos et al., 2011 [28]	423	**Spain**	75+ subgroup	Not available	Cross-sectional study	Demand for instructors and instructor effort as a barrier to PA	• 14% of participants found instructor performance a barrier to PA
Jefferis et al., 2014 [29]	1,680	**UK**, British Regional Heart Study	71–93 Mean 78.3 (SD 4.6)	0	Cross-sectional analysis of a cohort study	Falls history, fear of falling and associations with PA	• Having one fall in the previous 12 months (compared with no falls) was not associated with daily PA, but having two or more falls was 942 fewer steps compared with non-fallers, 95% CI 503–1,381) and 10 min/day less moderate-vigorous PA (95% CI 5–15) • Fear of falling is associated with 45 more sedentary minutes a day (95% CI 35–56) • In men who had a fear of falling, there was no evidence of a greater impact on PA levels among those who had fallen compared with those who had not fallen (*P* > 0.4)
Stathi et al., 2012 [54]	240	**UK**, Older People and Active Living (OPAL) study	70+	Not listed	Mixed methods study	Determinants of neighbourhood activity	• Health problems were listed as the most important barrier for men and women (mean score in men 3.9, in women 4.8), and fatigue as the second most important (men 3.7, women 4.1) • Lack of company was the third most important barrier for women, but only seventh for men • Environmental factors (e.g. lack of transport and lack of facilities) were found to be among the least important factors (means not given)
Tallis et al., 2022 [47]	96	**UK**	70+ Mean 74.8 (SD 4.4)	64	Longitudinal cohort study	Determinants of intended PA behaviour following easing of COVID-19 restrictions	• COM-B questionnaire showed: Capability (*n* = 229) and Motivation (*n* = 211) barriers were more commonly cited than Opportunity (*n* = 131) (*P* < 0.001) • Most frequently cited barriers were physical stamina (*n* = 51) and physical strength (*n* = 41)
							• Most frequently cited motivators were ‘care about the consequences of not doing it’ (*n* = 53) and develop habit for doing it (*n* = 53)
Yardley & Smith, 2002 [30]	224	**UK**, Wessex Fracture Prevention Trial	76–98 Mean 80.7 (SD 4.25)	52.7	Baseline data from RCT	Fear of falling and avoidance of activity	• Fear of falling and history of falling were predictive of the avoidance of activity in older adults measured using the SAFFE score at baseline and 6 months**
Clark, 1999 [31]	333	**USA**	70+	Subgroup: not available	Cross-sectional study	Measures of PA and perceived barriers	• Environmental barriers were most reported (87%), followed by physical symptoms (63%) • Most commonly mentioned symptoms were: pain, chest pain and shortness of breath • 37.2% of respondents reported doctor had discussed PA with them, but this was not related to number of weekly minutes of PA
Cohen-Mansfield et al., 2004 [55]	322	**USA**, Hebrew Home Study of Impairments and Exercise	74–85	58	Prospective cohort study	Barriers and motivators to PA	• Most important exercise attributes were quality of instructor (mean importance = 2.16/4) and type of exercise (mean importance = 2.03/4) • Wide variety in social exercise preferences: walking with a companion 33%, walk alone 18%, walk in a group 13%, indifferent to the social context of walking 36%
							• 31.2% reported a physician’s advice to exercise as very important • Social motivators were ranked as least important: 10th, 13th and 14th out of 14
Jerome et al., 2006 [41]	710	**USA**, Women’s Health and Ageing Studies I and II	70–79	100	Cross-sectional study	Functional deficits and their associations with PA	• Having any functional deficit was associated with being inactive (OR 6.54, 95% CI 3.01–14.18) • Having self-care and higher functioning care needs had an OR of 17.7 for being inactive compared with those with no functional deficit (*P* < 0.001)
Kahana, Kahana & Zhang, 2005 [37]	453	**USA**, Clearwater retirement study	72–98 Mean 79.13 (SD 4.13)	64.9	Prospective cohort study	Future orientation and its link to exercise	• Participants with the lowest future orientation (i.e. thought about the future the least) had a greater decline in time spent exercising compared with those with high future orientation (0.93 h/week per year compared with 0.57)
Li, Cardinal & Vuchinich, 2009 [38]	7,527	**USA**, Longitudinal Study of Ageing	70–99 Mean 76.83 (SD 5.59)	62	Cross-sectional study	Health worry and walking difficulty	• Health worry was negatively correlated to self-reported PA (B = -.24, *P* < 0.001)
Pascucci, Chu & Leasure, 2012 [56]	52	**USA**	80–101	76.9	Cross-sectional study	Factors and barriers contributing to health promotion	• Out of 18 barriers ‘impairment’ was the most recorded (stats not given) with ‘too tired’, ‘lack of transportation’ and ‘bad weather’ also frequently reported • ‘Lack of help from healthcare professionals’ was the least important barrier
Satariano, Haight & Tager, 2000 [57]	656	**USA**, Study of Physical Performance and Age-Related Changes in Sonomans	75+	59.8	Prospective cohort study	Reasons given for limitation or avoidance of PA	• Commonest reasons for women: fatigue (45.5%), concerns about falling (35.3%), arthritis (33.0%) • Commonest reasons in men: fatigue (33.7%), no interest (32.8%), other health problems (29.1%) • ‘need for a walking aid’ and ‘recommendation by physician’ were not in top 5 of 17 barriers enquired about for men or women

^*^P-E fit is a theoretical concept focusing on the interaction between the characteristics of an individual and the environment around them

^**^SAFFE score is a survey which assesses level of activity restriction in elderly individuals

We performed a narrative review of the evidence using the socioecological model as a theoretical framework to extract key themes [20]. Barriers and motivators were categorised as intrapersonal (physical or psychological), interpersonal or environmental, allowing assessment of the relative roles of individual, social and environmental factors.

### Risk of bias

All included studies were assessed by two of the team of reviewers independently (JH, MR, AK) using the Risk of Bias Assessment Tool Table 2 [21].

**Table 2:** Risk of bias of included studies using ROBANS tool

Reference	**Selection of participants**	**Confounding variables**	**Measurement of exposure**	**Blinding of outcome assessments**	**Incomplete outcome data**	**Selective outcome reporting**
**Aspvik et al.** [26]	-	/	-	-	/	-
**Benzinger et al.** [24]	-	+	-	-	-	-
**Booth, Bauman & Owen** [48]	-	-	-	-	-	-
**Brown & Miller** [49]	/	-	-	-	-	-
**Bruce, Devine & Prince** [50]	-	-	-	-	-	-
**Clark** [31]	-	-	-	-	-	-
**Cohen-Mansfield et al.** [55]	/	/	-	-	-	-
**Cousins** [35]	-	/	-	-	-	-
**Cousins** [34]	-	-	-	-	-	-
**De Roza et al.** [58].	/	-	-	-	-	-
**Eronen et al.** [45]	-	-	-	-	-	-
**Eronen et al.** [42]	-	-	-	-	-	-
**Ferrand, Martinent & Bonnefoy** [36]	/	-	-	-	-	-
**Hamm et al.** [33]	/	-	-	-	-	-
**Harada et al.** [25]	-	-	-	-	-	-
**Izquierdo Campos et al.** [28]	/	/	-	-	/	/
**Jefferis et al.** [29]	-	-	-	-	-	-
**Jerome et al.** [41]	-	-	-	-	-	-
**Kahana, Kahana & Zhang** [37]	/	-	-	-	-	-
**Keskinen et al.** [43]	/	/	-	-	-	-
**Keskinen et al.** [44]	/	/	-	-	-	-
**Kirkby et al.** [39]	-	-	-	-	-	-
**Li, Cardinal & Vuchinich** [38]	-	-	-	-	-	-
**Moschny et al.** [53]	-	-	-	-	-	-
**Newson & Kemps** [51]	-	/	-	-	/	-
**Pascucci, Chu & Leasure** [56]	/	/	-	-	/	-
**Portegijs et al.** [22]	-	-	-	-	-	-
**Rantakokko et al.** [46]	-	/	/	-	-	-
**Rantakokko et al.** [32]	-	/	/	-	-	-
**Rasinaho et al.** [52]	-	/	-	-	-	-
**Sakari et al.** [40]	-	-	-	-	-	/
**Satariano, Haight & Tager** [57]	-	/	-	-	/	/
**Sorensen & Gill** [27]	-	/	-	-	-	-
**Stathi et al.** [54]	/	/	-	-	-	-
**Tallis et al.** [47]	+	+	-	-	-	-
**Tsai et al.** [23]	/	/	-	-	-	-
**Yardley & Smith** [30]	-	+	-	-	/	-

Key: - = Low risk of bias, + = high risk of bias, /= unclear risk of bias

## Results

Overall, there were 26,961 (median 333, range 36–7,527) participants across the studies. Of the 37 included studies, 23 examined barriers, seven examined motivators and seven included both barriers and motivators. Most studies quantified the impact of specific barriers and motivators on PA level cross-sectionally (*n* = 24) [22–44], and/or longitudinally (*n* = 4) [32, 45–47]. PA level was measured by self-report (*n* = 29), accelerometer (*n* = 7) and Short Physical Performance Battery (SPPB) (*n* = 3). Other studies quantified the relative importance of different barriers and motivators as reported by the participants, with no measure of PA level [33, 48–57].

### Participant characteristics

A total of 10 studies were conducted in Finland, seven in the USA, five in Australia, four in the UK, three in Canada, two in Germany and Norway, and one each in France, Japan, Singapore and Spain (Table 1). Study participants’ age range was 70–101 years. A total of 21 studies gave a mean age (median 77.6 years, range 75.0–83.9) and two gave a median (77 and 83.5 years). Most studies were predominantly female (median 62.1% female, range 0–100); six studies included solely older women and one study solely older men. Data on gender were not available in five studies. Only seven studies reported ethnicity: six had predominantly white subjects (range 72–100%) and one was predominantly Chinese (75%). Only five studies reported socioeconomic status.

### Risk of bias

Risk of bias assessment is shown in Table 2. Overall, study quality was good with only three studies scoring a high risk of bias in any of the specific criteria. All three were due to high risk for confounding variable bias: one used a convenience sample of more physically active older adults [24], one comprised adults who self-selected to be included in a study investigating hip fracture prevention [30] and one recruited responders to an internet survey about PA [47]. For many of the cross-sectional studies, participant selection bias was unclear due to incomplete reporting of the participant recruitment process. Two of the studies were conducted during the COVID-19 pandemic which may make their findings less generalisable [47, 58].

## Barriers to PA (Table 3)

### Intrapersonal barriers: physical

#### Concerns about health and fitness

A total of 14 studies reported health and fitness as a barrier to PA, making it the most reported barrier [27, 31, 38, 40–42, 47, 48, 51–54, 56, 57]. Furthermore, in most studies where it was included it was the most important barrier. Concerns reported fell into three categories: (i) physical fitness was too poor to undertake PA; (ii) symptoms limited ability to undertake PA and (iii) PA could exacerbate health problems (most often pain, then shortness of breath) [31]. Experiencing health concerns as a barrier limited PA [38, 42, 52, 53].

**Table 3:** Summary of evidence for barriers affecting PA in older adults

**Potential barrier to PA**	**Number of studies**	**Li 2009**	**Jefferis 2014**	**Bruce 2002**	**Aspvik 2018**	**Eronen 2014b**	**Portegijs 2017**	**Sakari 2017**	**Jerome 2006**	**Sorensen 2007**	**Tsai 2013**	**Satariano 2000**	**Rasinaho 2007**	**Rantakokko 2010**	**Izquierdo Campos 2011**	**Brown 2001**	**Clark 1999**	**Cousins 1995**	**Cousins 1996**	**Moschny 2011**	**Stathi 2012**	**Yardley 2002**	**Rantakokko 2012**	**Keskinen 2020a**	**De Roza 2023**	**Booth 2002**	**Ferrand 2014**	**Tallis 2022**	**Newson 2007**	**Pascucci 2012**	**Benzinger 2014**
**n**		**7,527**	**1,680**	**1,500**	**1,219**	848	848	848	710	690	657	656	645	643	423	395	333	327	327	286	240	224	214	167	163	153	100	*96*	*94*	*52*	*36*
**Intrapersonal: physical**																															
Concerns about health/fitness	14	**+**				+		+	+	+		+	+				+			+	+					+		+	+	+	
Fear of falling/history of falls	11		**+**	**+**		+		+		+		+	+							+	+	+							+		
Fatigue	7					+				+		+	+				+				+									+	
Feeling too old	4					+		+													+					+					
Higher functioning and self-care abilities	1								+																						
Need for walking aid	1											+																			
Afraid of leaking urine whilst exercising	1															+															
**Intrapersonal: psychological**																															
Lack of motivation/interest	13					+		+		+		+	+				+		+	+	+					+	+		+	+	
Other psychological factors	7					+				+		+					+									+		+		+	
Lack of knowledge^a^	5							+					+				−											+		+	
Want more rest and relaxation time	2									+																+					
**Interpersonal**																															
Lack of company	9					+		+		+		+	+							+	+					+			+		
Lack of support by healthcare professional or family^b^	8					+		+				+	+				−	+	+											+	
Lack of time/caring role	8									+			+				+			+	+					+		+		+	
Performance of the sports instructor	1														+																
**Environmental**																															
Environmental barriers^c^	10					+	+	+			+		+	+			+						+	+							+/−
Safety	9					+		+				+	+	+			+								+				+	+	
Weather	7				+	+		+					+				+												+	+	
Lack of facilities	6												+							+	+					+			+	+	
Cost	5									+			+													+		+		+	
Transport	5									+										+	+								+	+	
Lack of equipment/opportunity^d^	3																			+						−		+			

**Bold studies indicate larger studies, with >1,000 participants**

Studies in italics indicate smaller studies, with <100 participants.

+Study asked about this barrier and found it to be present or associated with level of PA.

-Study asked about this barrier but did not find it to be present or associated with level of PA

Blank cells did not ask about this barrier.

Summary of **negative** findings as highlighted in the table above:

^a^Clark et al. found that a score on a quiz on exercise facts was not related to minutes of weekly PA undertaken.

^b^Clark et al. found that whether the doctor discussed PA with the patient or not had no effect on the number of minutes of weekly PA undertaken.

^c^Benzinger et al. found number of (but not presence of) environmental barriers was not associated with level of PA.

^d^Booth et al. found no respondents reported that lack of equipment or clothes was a barrier to PA.

Three studies found that ‘older old’ participants reported poor health as a barrier more often than the ‘young old’ [48, 50, 53], demonstrating that age was a modifying factor. Functional impairment was associated with being inactive [41]. However, use of a walking stick or aid was not rated as an important barrier, or predictive of being sedentary [50, 57].

#### Fear of falling/history of falls

All the studies which included falls and/or fear of falling found they were a barrier (*n* = 11) [27, 29, 30, 40, 42, 50–54, 57]. Fear of falls was a more significant barrier to PA than history of falling. A study of 1,500 women found fear of falls was negatively associated with being physically active, independent of history of falls in the previous 3 months [50]. In a UK study (*n* = 1,680), fear of falling was associated with being sedentary for an extra 45 min a day (95% CI 35–56) [29]. Those who had fallen zero or one times in the past year had the same PA level, but those who had fallen twice or more had lower PA levels, indicating that frequency of falling is important.

#### Fatigue

Fatigue was a barrier in all seven studies that asked about it [27, 31, 42, 52, 54, 56, 57]. One study found it was the most frequently reported reason [57], and in another the second commonest reason [54].

#### Feeling too old

Several studies found self-reporting feeling ‘too old’ to undertake PA was a barrier, with its importance increasing with higher age and poorer health status [40, 42, 48, 54].

### Intrapersonal barriers: psychological

#### Lack of motivation/interest

Lack of interest in exercise was found to be a barrier in 13 studies, although prevalence increased with poorer health, worse functional status and male gender [27, 31, 34, 36, 40, 42, 48, 51–54, 56, 57].

#### Other psychological factors

Seven studies included other psychological factors with mixed findings [27, 31, 42, 47, 48, 56, 57]. Two studies found not identifying as a ‘sporty person’ was important [27, 48], whereas another found that psychological capability barriers (e.g. mental stamina) were less important than physical capability barriers (e.g. physical strength) [47]. The following psychological barriers were not important: difficulty concentrating; lack of enjoyment of PA; and being too shy or embarrassed [48, 57].

#### Lack of knowledge

Lack of knowledge about exercise was not a significant barrier to PA [31, 40, 47, 52].

### Interpersonal barriers

#### Lack of company

Prevalence of lack of company as a barrier ranged from 1% to 43% across nine studies [27, 40, 42, 48, 51–54, 57]. Two studies reported it was a more important barrier for women than men [54, 57]. Interestingly, living alone increased the probability of meeting PA recommendations [46]. Those with an advanced walking limitation were five times more likely to report lack of company as a barrier than those with no walking limitations [40].

#### Lack of support by a healthcare professional, or family/friends

Lack of support by medical professionals was seen as relatively unimportant [31, 34, 40, 42, 52, 56, 57], with one study finding that whether the doctor discussed PA was not predictive of weekly number of minutes of PA [31]. Two studies found that relatives not wanting participants to exercise outside were not a significant barrier [40, 42].

#### Lack of time/caring role

Studies which asked whether lack of time was a barrier found it to be of low to moderate importance, and there were no recorded gender differences [48, 53, 54].

### Environmental barriers

#### Domestic and local area environmental barriers

Domestic and local area environmental barriers were included in 10 studies [22–24, 31, 32, 40, 42, 43, 46, 52]. The impact of barriers in the house or at the entrance to the house on PA level was mixed. In one study the odds of going outside less than daily increased with perceived entrance-related barriers (e.g. stairs) [22], but another study found they were not associated with level of PA [23].

Environmental barriers in the local area included perceived long distance to services [23]; hills in the surrounding area [46] and distance/lack of resting places [32]. Those with no mobility limitation reported environmental barriers most frequently, but in those with a mobility limitation they were the third most common [52].

#### Safety

All nine papers which included perceived safety as a potential barrier found it to be present [31, 40, 42, 46, 51, 52, 56–58]. Older adults who had high safety concerns (e.g. slippery roads) were five times more likely to not meet PA recommendations [42]. Safety concerns were also found to predict unmet PA need at 2 year follow-up [46]. Those with functional impairment were more likely to find safety issues a barrier [52].

#### Weather

Sakari *et al*. [40] found the most important barrier across all mobility levels was snow and ice, however Aspvik *et al*. [26] found that weather explained only 1.2% of the variance in recorded PA level.

#### Lack of facilities, equipment or opportunity

Lack of facilities, equipment and opportunity were found to be of moderate to low importance [47, 48, 51–54, 56]. In an Australian study, only 4.5% of the participants selected ‘no suitable facility nearby’, and no one selected ‘I have not got the right clothes or equipment’ as a barrier [48].

#### Transport and cost

Lack of transport was not an important barrier, but it was more often cited by women than men [53, 54]. Similarly cost was not an important barrier to most either: a Norwegian study found it to be the least important barrier [27]; only 6.6% selected it in an Australian study [48]; and only 6 out of 96 respondents cited finance as a barrier in a UK study [47].

### Motivators to PA (Table 4)

**Table 4:** Summary of evidence for motivators affecting PA in older adults

**Potential motivator to PA**	**Number of studies**	**Harada 2018**	**Keskinen 2020b**	**Sakari 2017**	**Rasinaho 2007**	**Kahana 2005**	**Cousins 1995**	**Cousins 1996**	**Cohen-Mansfield 2004**	**Eronen 2014a**	**Hamm 2014**	**Ferrand 2014**	**Tallis 2022**	**Newson 2007**	**Kirkby 1999**
**n**		**2,824**	848	848	645	453	327	327	322	261	107	100	*96*	*94*	*45*
**Intrapersonal: Physical**															
Disease prevention/Health management ^a^	4	**−**			+			−						+	
Fitness	2													+	+
Want to get rid of excess energy	2													+	+
**Intrapersonal: Psychological**															
Personal benefits/fulfilment	5				+							+	+	+	+
Motivation	3			+	+						+				
Stress relief	2													+	+
How often do you think about the future?	1					+									
Self-efficacy and Health locus of control	1							+							
**Interpersonal**															
Family and friends support	5						+	+					+	+	+
Social/company	5				+				+				+	+	+
Support from doctor/nurse/AHP	4						+	+	+					+	
Other participants similar age	1								+						
Quality of instructor and type of exercise	1								+						
**Environmental**															
Outside facilities	5		+	+	+				+	+					
Features of own home	2			+						+					
Familiar surroundings	1			+											
Suitable weather	1				+										
Financial factors	1								+						
I like to travel to the exercise sessions	1														+

**Bold studies indicate larger studies, with >1,000 participants**

Studies in italics indicate small studies, with <100 participants

+Study asked about this barrier and found it to be present or association with level of PA

-Study asked about this barrier but did not find it to be present or association with level of PA

Blank cells did not ask about this barrier.

^a^Summary of **negative** findings as highlighted in the table above:

• Harada et al. found that believing exercise to be a factor in dementia prevention was not associated with steps per day or time spent in moderate or vigorous activity.

• Cousins et al. found that motivation ‘to live a long and healthy life’ and the perceived risks and benefits to health of six specific exercises (e.g. brisk walking for 20 min) were not associated with weekly exercise status (*P* > 0.01).

#### Intrapersonal motivators: physical

Four studies included health-related motivators [25, 34, 51, 52] and two studies included fitness-related motivators [39, 51].

There was little evidence that the belief that undertaking PA would improve or maintain health led to increased PA. A study of Canadian women found that health incentive (i.e. desire to live a long and healthy life) was not associated with PA level [34], and a Japanese study found that 86% of participants believed exercise decreased the risk of developing dementia, but found no association between having this expectation and PA level [25]. Health maintenance was more important for individuals with no mobility limitation, and disease management was more important for those with limited mobility [52].

Wishing to improve or maintain fitness was found to be an important motivator, along with wishing to get rid of excess energy [39, 51].

#### Intrapersonal motivators: psychological

Personal benefit or fulfilment was found to be an important motivating factor [36, 39, 47, 51, 52], however one Australian study found that in the older group (75y+) personal fulfilment was less important than social motivators [39].

Intrinsic motivation (e.g. self-determined motivation) was found to be an important motivator [40, 52], and was a greater determinant of higher PA levels than extrinsic regulation (e.g. undertaking exercise for a reward) [36]. Stress relief was moderately important and was ranked equally important for older women (>75 years) and younger women (50–74 years) [39].

#### Interpersonal motivators

Three key subthemes were evident within the interpersonal theme: support from family and friends [34, 35, 39, 47, 51], opportunity to socialise and have company [39, 47, 51, 52, 55] and support from a healthcare professional [34, 35, 51, 55].

Two studies of older women found that support from family and friends was an important motivator [34, 35], but another study of older women [39], and two studies which included both men and women [47, 51], did not. Similarly, a study of older women found socialising an important motivator [39], whereas studies including men and women found it to be of moderate to low importance [47, 51, 52, 55].

Four studies found physician support to be an important motivator [34, 35, 51, 55], in contrast to the above section noting that the absence of a health practitioner discussing PA was not a barrier. This may indicate that where active support is motivating, the absence of support does not serve as a deterrent.

#### Environmental motivators

The five studies examining outside facilities found that having facilities or green spaces near home was a key motivator [40, 44, 45, 52, 55] and was associated with a decreased risk of developing walking difficulty at 3.5 year follow-up [45]. Features of participants’ own homes were also important [45], and having your own garden was perceived as a motivator by all walking abilities [40].

## Discussion

This systematic review identified 37 studies with quantitative data on barriers and motivators to PA in adults 70 and over. Studies identifying barriers were more common than studies on motivators. The most frequently identified barriers were (1) concerns about health and fitness (number of studies = 14, e.g. symptoms which prevented them undertaking PA, most notably pain), (2) lack of motivation and/or interest (*n* = 13), (3) fear of falling and history of falling (*n* = 11) and (4) environmental barriers (*n* = 10). The most frequently cited motivators were (1) support by family and friends (*n* = 5), (2) social aspects of PA (*n* = 5), (3) personal benefits (e.g. fulfilment) (*n* = 5) and (4) outside facilities (e.g. nearby green space) (*n* = 5).

The importance of the identified barriers and motivators varied across gender, age, geographical location and functional ability. There was evidence that lack of company, lack of transport, social support from friends and family and the social element of PA were more important to women, and that lack of interest/motivation was more important in men. Ethnicity was seldom reported and when it was the studies were predominantly white, so we are unable to comment about ethnic differences which are an important area for future research. The importance of poor health as a barrier increased with age, and the importance of weather varied by country. Those with functional impairment rated environmental and fear-based barriers more commonly, but also ranked ‘disease management’ more highly.

Some of the barriers and motivators identified are not easily modifiable but we have identified three key areas where action could be taken: (1) Healthcare providers should be supported and encouraged to advise older adults to undertake regular PA within their limits [34, 35, 55]. Most major guidelines state that exercise is generally safe for older people and they do not need to consult a medical practitioner before undertaking increased levels of PA [6, 59]. (2) Healthcare providers should be aware that fear of falling is common in older adults, and should stress during fall-related consultations or at falls prevention classes that PA is one of the most effective ways of reducing incidence of falls in this age group [60]. (3) At a societal level, architects, town planners and local authorities should consider ease of access to facilities for PA for older adults, and safety considerations (e.g. street lighting) early in the design process [40, 44, 45, 52, 55].

We found overlap between the findings from the qualitative arm of our systematic review [61] and this arm. The qualitative paper provides in-depth accounts of people's subjective experiences and factors that are meaningful to them, and the quantitative paper provides effect sizes and evidence of impact on PA levels. Together they provide a holistic account of the evidence. Both studies found barriers relating to physical health and fitness concerns, risk of injury (including fear of falls) and environmental barriers in the surrounding area, and motivators relating to social interaction and positive experiences (e.g. enjoyment). The qualitative paper highlighted the damaging impact of negative ageing stereotypes and self-stigma on older adult's participation in PA, consistent with the quantitative findings regarding feeling 'too old' to be active. However, there were areas where the findings between our two reviews differ. The qualitative arm found that health gains were a significant motivator, whereas in this paper we found that believing that PA will improve health did not actually impact on PA level [25, 34]. The qualitative review found that older adults consider weather to be an important barrier, but we found the actual impact on PA was small (1.2% of the variance) [26]. Therefore, the factors older adults think are the most important may not actually impact upon PA level the most.

A previous mixed-methods systematic review from 2011 included any study with at least two participants over the age of 79 years (range 19–108), chosen as their scoping review had identified a dearth of studies solely of older adults [13]. We identified 18 studies which have been published since this review, highlighting that older adults are increasingly being included in this field. They found health status to be the most cited barrier, the same as our review, and that participant fear was an important barrier. They grouped fear into one subcategory which included fears about safety, falling and injury, whereas we have reviewed these areas separately to allow a fuller understanding. Another mixed-methods systematic review from 2019 (50–70 years) included only six quantitative studies, but 49 qualitative studies, whereas we identified 37 quantitative studies in those ≥70 years [12]. The three most common barriers in the 65–70 year subgroup were environmental, beliefs about capabilities (including health concerns) and social influences. However, unlike our review, they did not identify lack of motivation/interest, or history or fear of falling as important barriers. This may reflect the younger age group they were studying. The three most common motivators were social influences, reinforcement (e.g. pleasure) and behavioural regulation (e.g. advice from health professionals), which were similar to our findings. However, they found access to facilities was unimportant, whereas it was a top motivator in our study.

### Strengths and weaknesses

To our knowledge, this is the first systematic review of the quantitative evidence for barriers and motivators for PA to solely include adults over 70 which did not focus on a specific disease or demographic. The application of the socioecological model allowed us to extract key themes aligned to a well-established theoretical framework. This should allow comparison with aligned and future reviews in this area. This paper in conjunction with our parallel qualitative review gives a comprehensive overview of the evidence in this area, with the complementary methodologies ensuring all important barriers and motivators were included.

We were unable to synthesise the data numerically, however if future studies include barriers and motivators which are sufficiently homogenous in design then meta-analysis may be possible. Some barriers and motivators were only included in one study; therefore, a lack of evidence may not indicate that a factor is unimportant, just that there is an absence of evidence. Very few factors were found *not* to be a barrier or motivator when included in a study (see Tables 3 and 4), indicating that if the researcher asks specifically about a factor at least some respondents are likely to select it. This may have led to some factors being over or under emphasised in this review purely as a function of how often the included studies asked about them.

Only two of the included studies had a mean or median ≥81 years, and with life expectancy continuing to rise it will be important to investigate barriers and motivators in the oldest old (i.e. 85+ years). All the studies we identified were from high-income countries, and only one study had a recorded ethnicity that was not predominantly white. Future research in this area should aim to include older, frail participants, and those from a wider demographic base to ensure results are applicable to patient populations across the world.

### Implications for policy and future research

This review has identified three key modifiable areas which could be targeted to increase levels of PA in adults over 70: the role of health professionals in promoting PA; targetting those with fear of falling with education and support; and prioritising ease of access to facilities for PA and safety when developing new housing or town planning. These factors were identified across studies and are therefore likely to be reproducible in different countries and settings, although further work in low- and middle-income countries and with greater ethnic diversity is required. We hope that policy can now be used to address these modifiable areas to enable older adults to take up and maintain recommended levels of PA.

## Supplementary Material

aa-23-1346-File002_afae080
