# Correction to: Barriers and motivators to undertaking physical activity in adults over 70—a systematic review of the quantitative literature

**DOI:** 10.1093/ageing/afae112

**Published:** 2024-06-05

This is a correction to: Alixe H M Kilgour, Matthew Rutherford, Joanna Higson, Samantha J Meredith, Jessica McNiff, Stephanie Mitchell, Anusan Wijayendran, Stephen E R Lim, Susan D Shenkin, Barriers and motivators to undertaking physical activity in adults over 70—a systematic review of the quantitative literature, Age and Ageing, Volume 53, Issue 4, April 2024, afae080, https://doi.org/10.1093/ageing/afae080

The originally published version of this article has been corrected.

In the legends of Table 3 and Table 4, the sentence “Studies in grey indicate smaller studies, with <100 participants.” has been corrected to read “Studies in italics indicate small studies, with <100 participants.”.

In addition, the formatting of headings in the Results section has been corrected.

The Publisher apologizes for not correcting these errors during an earlier production stage.

