# Supplementary material for: Barriers and motivators to undertaking physical activity in adults over 70—a systematic review of the quantitative literature
Source: Age Ageing. 2024 Apr 22;53(4):afae080. doi: 10.1093/ageing/afae080 (PMC11036106; doi:10.1093/ageing/afae080)
Supplement: aa-23-1346-File002_afae080 [file aa-23-1346-file002_afae080.docx]

### Barriers and Motivators to Undertaking Physical Activity in Adults over 70 – A Systematic Review of the Quantitative Literature

## Appendix 1- Database Search Strategies

**CINAHL**

1. Barrier*
2. MH “motivation+”
3. Motivat*
4. Facilitat*
5. Inhibit*
6. MH “exercise+”
7. MH “recreation+”
8. MH “physical fitness+”
9. MH “Sports+”
10. MH “Physical Activity”
11. MH “Aged”
12. MH “Aged, 80 and Over”
13. MH “Frail Elderly”
14. Old*
15. Elder*
16. 1 or 2 or 3 or 4 or 5
17. 6 or 7 or 8 or 9 or 10
18. 11 or 12 or 13
19. 16 and 17 and 18

**Embase**

1. Barrier*
2. exp motivation/
3. Motivat*
4. Facilitat*
5. Inhibit*
6. exp physical activity/
7. exp exercise/
8. exp sport/
9. exp fitness/
10. old*
11. aged/ or frail elderly/ or very elderly/
12. Elder*
13. 1 or 2 or 3 or 4 or 5
14. 6 or 7 or 8 or 9
15. 10 or 11 or 12
16. 13 and 14 and 15

**Medline**

1. Barrier*
2. exp motivation/
3. Motivat*
4. Facilitat*
5. Inhibit*
6. physical activity*
7. physical exertion/
8. exp physical fitness/
9. exp exercise/
10. exp recreation/
11. exp aged/
12. elder*
13. old*
14. 1 or 2 or 3 or 4 or 5
15. 6 or 7 or 8 or 9 or 10
16. 11 or 12 or 13
17. 14 and 15 and 16

**PsycINFO**

1. Barrier*
2. exp motivation/
3. Motivat*
4. Facilitat*
5. Inhibit*
6. exp physical activity/
7. exp exercise/
8. exp recreation/
9. physical fitness/
10. old*
11. elder*
12. exp gerontology/ or exp aging or exp geriatrics/
13. exp health impairments/
14. 1 or 2 or 3 or 4 or 5
15. 6 or 7 or 8 or 9
16. 10 or 11 or 12
17. 14 and 15 and 16

**Web of Science (Science Citation Index Expanded 1900-present, all searches in TOPIC dropdown menu)**

1. Barrier*
2. Motivat*
3. Sport*
4. Exercis*
5. Fitness
6. Recreation
7. Walking
8. Older-adult
9. Frail*
10. 1 or 2
11. 3 or 4 or 5 or 6 or 7
12. 8 or 9 or 10
13. 11 and 12 and 13

**ASSIA**

(mainsubject.Exact("barriers" OR "facilitators" OR "motivation") OR inhib* OR motivat*)

AND

mainsubject.Exact("aerobic exercise" OR "sports" OR "fitness" OR "walking" OR "recreation" OR "aerobic fitness" OR "exercise" OR "physical activity")

AND

(mainsubject.Exact("frailty" OR "frail elderly" OR "aged, 80 & over" OR "frail elderly people" OR "elderly" OR "aged" OR "elderly people" OR "frail") OR elder*)

## Appendix 2- Data Extraction Sheet

**Data Extraction Extracted by:**

Study Information:

| **Study Number** |  |
| --- | --- |
| **Date Extracted** |  |
| **Title** |  |
| **First Author** |  |
| **Journal** |  |
| **Date Published** |  |
| **Country of origin (and language if not English)** |  |
| **Source of Funding** |  |
|  |  |

Study Characteristics:

| **Aim of study** |  |
| --- | --- |
| **Type of Study** | Cohort (Prospective)  Cohort (Retrospective)  Cross-Sectional  Case-Control  Other (specify) |
| **What was examined?** | **Motivators** – Yes/No  **Barriers** – Yes/No |

Participant Characteristics:

| **Sample Size** | Number approached -  Final number included in study – |
| --- | --- |
| **Age Range (Years) e.g. 73-94**  **Other differential (mean/median/IQ range etc)** | Whole study-  Subgroup relevant to this study (if different)- |
| **Gender, %male** |  |
| **Who was data collected from?** | Older People  Family members  Healthcare professionals |
| **Study Population (city-wide, walking group, exercise class etc)** |  |
| **Ethnicity (% as listed in study)** |  |
| **Socioeconomic Group** |  |
| **How participants recruited e.g. telephone, postal advert** |  |
| **Part of a larger study? Give details** |  |
| **Method of data collection** | Questionnaire  Interviews  Combination  Other (specify) |
| **Method of Data Analysis** | Quantitative  Mixed Qualitative and Quantitative |

Outcomes

| **Key findings reported** | |
| --- | --- |
| - **Motivators:** |  |
| - **Barriers:** |  |
| **Additional findings reported** |  |
| **Did the paper identify and consider confounding variables? (specify)** |  |
| **Separate comment on frail population?** |  |

Risk of bias (ROBANS)

| **Domain** | **Details** | **Risk of bias** |
| --- | --- | --- |
| 1. **Selection of participants** | Selection bias caused by the inadequate selection of participants | High/low/unclear |
| 1. **Confounding variables** | Selection bias caused by the inadequate confirmation and consideration of confounding variable | High/low/unclear |
| 1. **Measurement of exposure** | Performance bias caused by the inadequate measurement of exposure | High/low/unclear |
| 1. **Blinding of outcome assessments** | Detection bias caused by the inadequate blinding of outcome assessments | High/low/unclear |
| 1. **Incomplete outcome data** | Attrition bias caused by the inadequate handling of incomplete outcome data | High/low/unclear |
| 1. **Selective outcome reporting** | Reporting bias caused by the selective reporting of outcomes | High/low/unclear |
